# NAVIGATE‐AI Nursing Framework: A Scoping Review and Preliminary Expert Content Evaluation for Safe Bedside AI Use in Nursing

**DOI:** 10.1155/jonm/6525145

**Published:** 2026-10-09

**Authors:** Omar Alqaisi, Safia Darwish, Mohammed Dibas, Suhair Al-Ghabeesh

**Affiliations:** ^1^ Nursing Department Al-Zaytoonah University Airport Street Amman Jordan zuj.edu.jo; ^2^ Nursing Program Batterjee Medical College Jeddah 21442 Saudi Arabia bmc.edu.sa; ^3^ Faculty of Nursing Al-Zaytoonah University Airport Street Amman Jordan zuj.edu.jo; ^4^ Department of Medicine Faculty of Medicine and Health Sciences An-Najah National University Nablus State of Palestine najah.edu

**Keywords:** artificial intelligence, clinical decision-making, nursing practice, patient safety, scoping review

## Abstract

**Aim:**

To synthesize evidence on artificial intelligence (AI) applications in nursing clinical practice and to develop and evaluate the content relevance of an evidence‐informed, bedside‐oriented mnemonic framework supporting nurses’ safe and critical use of AI‐supported recommendations.

**Background:**

AI is increasingly integrated into nursing clinical practice, yet bedside nurses require concise guidance to appraise and use AI‐supported recommendations safely and ethically. Existing frameworks address organizational adoption or AI literacy education; a point‐of‐care cognitive aid for critical appraisal of AI outputs remains underdeveloped.

**Evaluation:**

A scoping review, reported in accordance with PRISMA‐ScR, searched PubMed/MEDLINE, CINAHL, Scopus, ScienceDirect, and Web of Science for English‐language publications from January 2015 to September 2026. Fourteen eligible sources were synthesized into five themes. An evidence‐informed seven‐component bedside cognitive guide was subsequently developed and evaluated by a purposively selected panel of 31 nursing and healthcare professionals using item‐ and scale‐level content validity indices.

**Key Issue:**

Relevance I‐CVI values ranged from 0.68 to 0.94, and the S‐CVI/Ave was 0.78, below the prespecified reference threshold of 0.90. These findings provide preliminary evidence regarding the relevance, clarity, and perceived bedside applicability of the framework components, but do not establish scale‐level content validity.

**Conclusion:**

The framework is an evidence‐informed cognitive aid, not a validated clinical decision rule. Further iterative expert consensus and prospective usability, feasibility, and implementation testing are required before broad application.

**Implications for Nursing Management:**

Nurse managers and health‐system leaders should prioritize structured AI‐literacy education, clear governance, and escalation pathways for AI‐supported recommendations, nurse involvement in AI procurement and implementation, and monitoring mechanisms for AI‐related safety events to ensure safe AI integration into nursing practice.

## 1. Introduction

Artificial intelligence (AI), encompassing machine learning, natural language processing (NLP), deep learning, and predictive analytics, is rapidly transforming healthcare delivery by enabling computers to perform cognitively complex tasks that historically required human expertise [1, 2]. Within nursing, AI has begun reshaping clinical practice across multiple domains, including real‐time patient monitoring, clinical decision support, medication safety, administrative documentation, and workforce management [3, 4]. As the largest professional group in the global health workforce and the primary interface between healthcare systems and patients, nurses are directly implicated by these developments, with profound implications for patient safety, care quality, and professional identity worldwide [5, 6].

A growing body of evidence has mapped AI applications across diverse nursing settings. Systematic and scoping reviews have confirmed AI’s potential in clinical decision support, early deterioration detection, predictive analytics for pressure injuries and sepsis, and workflow optimization [2, 3, 5]. In critical care, Park et al. [7] identified six distinct AI application domains, with predictive analytics and patient monitoring emerging as the most prevalent. Alqaraleh et al. [8] demonstrated that AI‐powered clinical decision support systems reduced medication errors by up to 95% in controlled surgical settings, though generalizability across diverse nursing environments remains limited, while Mikkonen et al. [9] confirmed AI’s capacity to improve clinical decision‐making outcomes. Notwithstanding these gains, both El Arab et al. [6, 10] and Rony et al. [2] cautioned that most reported benefits remain contextual and have not yet been universally scaled.

Despite this promising evidence base, the successful bedside implementation of AI remains constrained by significant barriers. Namdar Areshtanab et al. [11] reported that 41.1% of nurses exhibited low AI knowledge, while Taie and Ali [12] found that 58% of nursing personnel had unsatisfactory AI literacy, and none had received formal AI training. Bodur et al. [13] identified substantive concerns regarding algorithmic transparency, accountability, and the erosion of critical thinking through AI over‐reliance. Mohammed et al. [14] confirmed that existing regulatory and ethical frameworks are inadequately tailored to nursing‐specific AI contexts, and Wei et al. [1] underscored persistent technical barriers, including data heterogeneity, interoperability failures, and the black‐box opacity of advanced AI systems.

Against this backdrop, El Arab et al. proposed the Nursing AI Integration Roadmap (NAIIR), a six‐pillar framework for organizational‐level AI adoption [10]. However, organizational implementation frameworks do not necessarily provide a concise point‐of‐care cognitive aid for bedside nurses who must interpret, question, and apply AI‐supported recommendations during routine clinical care. Therefore, this study sought to systematically map evidence on artificial intelligence in nursing clinical practice and to translate evidence‐derived principles into the NAVIGATE‐AI nursing framework. This framework serves as an evidence‐informed, bedside‐oriented cognitive guide intended to support nurses’ critical appraisal and safe use of AI‐supported recommendations; it is not a substitute for clinical judgment, institutional governance, local policy, or validated clinical decision rules.

Accordingly, this study had three objectives: (1) to synthesize evidence on AI applications, nurse‐AI interactions, and implementation challenges in nursing clinical practice; (2) to develop an evidence‐informed, bedside‐oriented NAVIGATE‐AI nursing framework from the thematic synthesis; and (3) to evaluate the content relevance of the framework through structured expert review.

## 2. Materials and Methods

### 2.1. Study Design and Protocol

This study employed a scoping review design to systematically map the evidence regarding AI applications and integration in nursing clinical practice. The methodology followed the framework developed by Arksey and O’Malley [15], with methodological refinements from Levac et al.’s study [16]. The report adheres to the Preferred Reporting Items for Systematic Reviews and Meta‐Analyses extension for Scoping Reviews (PRISMA‐ScR) guidelines [17]. The guiding research question was as follows: “what is the current state of evidence on AI applications in nursing clinical practice and what principles should underpin a mnemonic framework for safe bedside implementation?” Consistent with PRISMA‐ScR methodology, a formal risk‐of‐bias assessment was not performed, as the objective was to map the breadth and nature of available evidence rather than synthesize effect sizes. The protocol was registered retrospectively on INPLASY (INPLASY202650075) on 13 May 2026, after literature searching, screening, and data charting had commenced. The scoping review and framework‐development activities were analytically distinct. The scoping review mapped the breadth and characteristics of available evidence. Framework development was subsequently conducted as a knowledge‐translation activity in which evidence‐derived themes and principles were organized into a bedside‐oriented mnemonic. The subsequent expert content‐evaluation exercise assessed the relevance of the proposed framework components; it was not part of the scoping‐review selection or synthesis process.

### 2.2. Protocol Deviations and Amendments

Because registration occurred after review activities had commenced, the registered protocol did not prospectively govern all final methods. First, narrative reviews were included in the final eligibility criteria to capture conceptual and practice‐oriented evidence relevant to nursing AI. Second, exclusion criteria were operationalized more explicitly during full‐text screening to exclude editorials, opinions, and conference abstracts. Third, the expert content‐evaluation component was added after registration as a subsequent knowledge‐translation activity and did not alter search, screening, or synthesis procedures. Fourth, gray literature searches and citation tracking were used solely for contextual identification and were not included in the formal synthesis.

### 2.3. Search Strategy

A comprehensive literature search was conducted in PubMed/MEDLINE, CINAHL, Scopus, ScienceDirect, and Web of Science. The search covered English‐language publications from 1 January 2015 to 5 September 2026. The search was initially conducted in March 2026 and updated on 5 September 2026. Search terms combined controlled vocabulary, where available, and free‐text terms related to artificial intelligence, machine learning, deep learning, natural language processing, clinical decision support, predictive analytics, nursing, clinical practice, implementation, integration, patient safety, knowledge, attitudes, ethics, and frameworks. Complete database‐specific search strings and final search dates are provided in Supporting Table S1.

Organizational website searches of the World Health Organization, the International Council of Nurses, and the American Nurses Association were conducted during the initial search process and updated on 5 September 2026. These searches combined artificial intelligence, nursing, ethics, competency, guidance, and patient safety. Backward reference‐list screening and forward citation tracking in Google Scholar and Web of Science were also conducted. Organizational materials and citation‐tracking outputs were used for contextual mapping and identification of potentially relevant peer‐reviewed publications; gray‐literature documents themselves were not eligible for the formal evidence synthesis. No additional peer‐reviewed publications meeting the prespecified inclusion criteria were identified through the search update.

### 2.4. Eligibility Criteria

Studies were eligible for inclusion if they (1) focused on artificial intelligence applications, integration, implementation, governance, or use in nursing clinical practice; (2) involved registered nurses, advanced practice nurses, nursing students, or nursing professionals as primary participants, users, or subjects; (3) reported on artificial intelligence applications in direct patient care, nurses’ knowledge, attitudes, acceptance, barriers or facilitators to adoption, ethical or regulatory considerations, or nursing‐specific artificial intelligence frameworks; (4) were published in English; and (5) were peer‐reviewed original research studies or peer‐reviewed evidence syntheses, including systematic reviews, scoping reviews, rapid reviews, integrative reviews, umbrella reviews, and narrative reviews. Gray literature searches of selected organizational websites were conducted to identify potentially relevant guidance documents and support citation tracking. However, because the formal eligibility criteria required peer‐reviewed original research or peer‐reviewed evidence syntheses, gray‐literature documents were not included in the final evidence synthesis or framework derivation.

Studies were excluded if they (1) focused exclusively on artificial intelligence in non‐nursing healthcare domains; (2) were editorials, opinion pieces, commentaries, conference abstracts, letters, or protocols without empirical data or substantive evidence synthesis; or (3) were published in languages other than English.

A broad range of peer‐reviewed primary and secondary evidence was intentionally included because the review question sought to map the breadth of nursing‐related artificial intelligence applications, nurse–AI interactions, implementation challenges, and practice‐oriented principles. Evidence type was retained during charting and synthesis to avoid conflating primary empirical findings with conclusions reported in secondary reviews.

### 2.5. Study Selection and Data Extraction

All retrieved citations were imported into EndNote 21 and deduplicated before screening. Two independent reviewers (O.A. and S.D.) screened titles and abstracts, followed by full‐text assessment of potentially eligible reports. Discrepancies were resolved through structured discussion, with a third reviewer serving as arbitrator when consensus could not be reached. The selection process is detailed in the PRISMA‐ScR flow diagram.

A standardized data charting form was developed a priori and piloted on two randomly selected studies before full extraction to ensure consistency. Data were charted independently by both reviewers, and discrepancies were resolved by consensus. Extracted fields included author(s), year, country, study design, population, AI technology examined, clinical setting, key findings, and reported outcomes. Data were synthesized using an inductive thematic analysis approach, wherein recurring patterns were identified, organized into preliminary codes, and clustered into broader themes through iterative discussion. To ensure trustworthiness, the following measures were applied: (1) independent coding by two reviewers with comparison before theme finalization (dependability); (2) consensus through documented iterative discussion sessions (confirmability); (3) consistent cross‐referencing of findings against source texts (credibility); and (4) consideration of the review’s geographic and design diversity when assessing transferability.

### 2.6. Framework Development

The NAVIGATE‐AI nursing framework was developed after the completion of the scoping‐review synthesis and was analytically distinct from study selection, data charting, and thematic synthesis. Framework development was undertaken as an evidence‐informed knowledge‐translation activity and not as a clinical evaluation exercise.

The development process comprised four stages. First, two reviewers identified recurring findings regarding the safe, effective, ethical, and contextually appropriate use of artificial intelligence in nursing through an inductive thematic synthesis. Second, each theme was translated into one or more practice principles. Third, candidate framework domains were generated by comparing these principles with the nursing artificial intelligence competency literature, patient safety principles, and healthcare artificial intelligence ethics guidance [3–6, 10, 14]. Fourth, the research team reviewed the candidate domains iteratively to identify conceptual overlap, consolidate closely related concepts, and select concise wording that could be organized into a memorable bedside‐oriented mnemonic.

To improve transparency and traceability, an evidence‐to‐framework derivation matrix is provided in Supporting Table S2. The matrix documents the relationship between thematic findings, extracted practice principles, supporting sources, candidate domains, rationales for consolidation, final NAVIGATE‐AI components, and wording refinements following expert feedback. No framework component was based on a single source; each component was informed by convergent findings across multiple included sources. The framework was subsequently subjected to an initial expert content evaluation. The questionnaire instrument is provided in Supporting Table S3.

### 2.7. Expert Content Evaluation

A purposive sampling strategy was used to recruit nursing and healthcare professionals with relevant experience. Eligible participants were required to report at least 1 year of experience in clinical practice, academia, informatics, patient safety, ethics, artificial intelligence, nursing education, or nursing research. This experience had to be relevant to the use, evaluation, or governance of artificial intelligence in healthcare or to education about its use. Potential participants were approached through professional nursing and academic networks and were invited to complete an anonymous online questionnaire. The invitation described the study purpose, the voluntary nature of participation, and the estimated completion time. Because the invitation link could be shared and the number of individuals who viewed the invitation was not recorded, a conventional response rate could not be calculated. Of the 31 analyzable respondents, 8 (25.8%) reported expertise in artificial intelligence in healthcare, and 10 (32.3%) reported expertise in nursing informatics. These groups were treated as a combined direct AI/informatics‐expertise subgroup for the sensitivity analysis. At the time of questionnaire administration, the draft framework was labeled “NURSE‐AI.” Following the identification of a separate 2026 publication using the same name, the framework was renamed NAVIGATE‐AI before manuscript submission to avoid confusion. This change affected the framework name only; the seven components, their order, questionnaire items, and expert ratings were unchanged.

Experts reviewed the seven NAVIGATE‐AI components and rated each component’s relevance to the safe implementation of bedside artificial intelligence in nursing using a four‐point ordinal scale: 1 = not relevant, 2 = somewhat relevant, 3 = relevant, and 4 = highly relevant. Ratings of wording clarity and perceived bedside applicability were also collected. Optional free‐text comments were reviewed descriptively and used to guide wording refinement; they were not subjected to a formal qualitative thematic analysis.

The item‐level content validity index (I‐CVI) was calculated as the proportion of experts assigning a relevance rating of 3 or 4 to each component. The scale‐level content validity index using the average method (S‐CVI/Ave) was calculated as the mean of the seven I‐CVI values. An I‐CVI of at least 0.78 and an S‐CVI/Ave of at least 0.90 were used as prespecified reference thresholds for interpretation. These indices were used to assess preliminary content relevance and did not establish clinical validity, usability, feasibility, implementation effectiveness, or patient benefit.

Thirty‐two questionnaires were received. One duplicate response was excluded before analysis, resulting in 31 analyzable responses. A conventional response rate could not be calculated because the total number of professionals who received or accessed the questionnaire link was not recorded. The responses were anonymous; consequently, the research team could not determine whether any individual respondent had prior involvement in framework development.

## 3. Results

### 3.1. Study Selection

The database search identified 1180 records across five electronic databases. Supplementary searches identified 27 records from organizational gray literature and 34 through backward and forward citation tracking, yielding 1241 records in total. After 356 duplicates were removed, 885 titles and abstracts were screened, and 690 were excluded. Full‐text retrieval was attempted for 195 reports; 10 were unobtainable. Of the 185 full‐text articles evaluated, 171 were excluded: not centered on nursing (*n* = 72), lacking direct AI clinical implementation (*n* = 51), ineligible publication types (*n* = 28), and nonrelevant outcomes (*n* = 20). Fourteen studies satisfied all inclusion criteria and were incorporated into the final synthesis (Figure 1).

**FIGURE 1:**
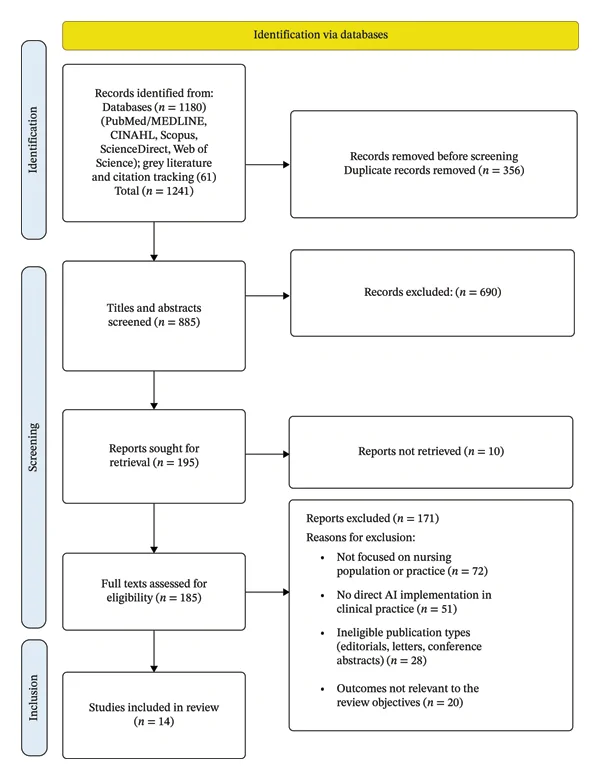
PRISMA flow diagram of study selection.

### 3.2. Overview of Included Studies

The 14 included sources, published between 2021 and 2026, represented diverse study designs, geographic contexts, and domains of artificial intelligence applications. The evidence base comprised 11 secondary evidence syntheses (two umbrella reviews, four systematic reviews, two scoping reviews, one integrative review, one rapid review, and one narrative review) and three primary studies (one descriptive correlational study, one qualitative descriptive study, and one descriptive cross‐sectional study). This heterogeneous evidence architecture was retained because the scoping review aimed to map the breadth of nursing‐related artificial intelligence evidence, rather than to estimate pooled effects.

Several included evidence syntheses may have included overlapping primary studies. Therefore, the synthesis was conducted at the level of recurring principles and themes, rather than by aggregating study counts or effect estimates across reviews. The reported number of primary studies cited in the included reviews should not be interpreted as an independent measure of evidence. Collectively, the included sources drew on substantial bodies of primary research and reported findings from diverse nursing settings. However, because multiple reviews may have synthesized some of the same primary studies, no cumulative estimate of independent primary‐study evidence was calculated. The framework should therefore be interpreted as an evidence‐informed knowledge‐translation product rather than as an evidence‐graded or quantitatively derived clinical tool. Table 1 summarizes all 14 studies and their connections to the NAVIGATE‐AI nursing framework components.

**TABLE 1:** Characteristics of the included studies (*n* = 14) and their relevance to the NAVIGATE‐AI Nursing Framework.

Author, year	Country	Study design	Evidence level	Sample size	AI type/application	Clinical setting	Key findings	Relevance to NAVIGATE‐AI Nursing Framework
Wei et al. [1]	China/multinational	Narrative review	Secondary evidence	Multiple studies	CDSS, patient monitoring, NLP, robotics, adaptive learning	Multiple nursing settings	AI advances clinical decision support, monitoring, workflow optimization, and education; barriers include technical constraints, ethical dilemmas, and workforce gaps; nursing‐specific tools and ethical frameworks are absent	A: clinical judgment must guide AI application; I: workforce adaptation and AI education are prerequisites for safe integration
Khan Rony et al. [2]	Multinational	Umbrella review (13 systematic reviews)	Secondary evidence	13 reviews synthesized	Multiple AI types (CDSS, predictive analytics, NLP, robotics)	Nursing practice (general)	AI enhances clinical decision support, reduces workload, and improves care quality; nurses require AI literacy and ethical competency; a gap persists in nursing‐specific AI tools	N: nurses must critically navigate AI‐generated outputs; A: AI should augment, not replace, clinical nursing judgment
von Gerich et al. [3]	Multinational	Scoping review	Secondary evidence	93 studies	AI‐based technologies (ML, NLP, expert systems)	Clinical nursing practice (multiple settings)	Most studies were in the developmental phase; only 7% tested in real clinical settings; 36.6% lacked ethical discussion; nurses largely excluded from AI design	E: the current AI evidence base is immature; nurses must evaluate quality before applying; R: ethical gaps in AI development require nurse vigilance
Seibert et al. [4]	Multinational	Rapid review	Secondary evidence	292 publications	ML, expert systems, NLP, robotics	Multiple nursing settings (hospital, community)	83.2% of AI applications were basic or experimental; few real‐world studies existed; ethical, legal, and social implications rarely addressed in nursing AI research	E: most AI evidence is experimental; bedside nurses must critically evaluate applicability; R: ethical and legal dimensions of AI are underaddressed in current nursing literature
O’Connor et al. [5]	Multinational	Systematic review	Secondary evidence	140 studies	Multiple AI types (diagnostic, monitoring, decision support)	Nursing and midwifery	Only 7.14% of AI studies demonstrated real‐world clinical benefit; AI literacy deficit among nurses; privacy, bias, and accountability risks identified	E: nurses must critically evaluate AI evidence before bedside application; R: accountability and bias require ethical recognition
El Arab et al. [6]	Multinational	Umbrella review	Secondary evidence	18 systematic reviews	Multiple AI types (education, clinical, operational)	Nursing practice and education (global)	Three themes: ethical implications, transformation of nursing education, strategies for AI integration; need for AI literacy frameworks and ethical guidelines; NAIIR framework expanded	I: AI literacy and structured integration strategies are foundational for safe practice; R: ethical implications of AI must be embedded in nursing education and clinical governance
Park et al. [7]	Republic of Korea	Scoping review	Secondary evidence	35 studies	Predictive analytics, patient monitoring, early warning systems	ICU/critical care settings	Six AI application domains identified; predictive analytics most prevalent; specialized narrow‐focus AI tools outperform general tools; limited real‐world evaluation	U: understanding AI tool scope and limitations is essential in high‐acuity settings; S: AI monitoring requires nurse oversight to safeguard patient safety
Alqaraleh et al. [8]	Jordan/multinational	Systematic review	Secondary evidence	12 studies	CDSS, smart infusion pumps, medication safety AI	Hospital (OR, ICU, medication management)	CDSS reduced OR medication errors by up to 95%; smart pumps reduced IV errors by 80%; 62% of nurses uncertain about trusting AI recommendations	S: AI significantly safeguards patient safety in medication management; E: despite efficacy data, nurses must evaluate AI recommendations before acting
Mikkonen et al. [9]	Finland/multinational	Systematic review (8 interventional studies)	Secondary evidence	1173 nurses and healthcare staff	AI‐supported clinical decision‐making tools (CDSS, ML‐based systems)	Hospitals (ICU, acute care, ED, NICU; USA, Korea, Canada)	AI significantly supports clinical decision‐making (reduced readmissions, ICU transfers, protocol deviations); specialized tools outperform generalized ones; usability and workflow integration are critical determinants; geographic bias (predominantly US)	N: nurses must critically navigate AI outputs and distinguish between specialized and generalized tools; A: AI decision‐support tools must align with nursing clinical workflows to be safely applied; S: workflow‐integrated AI tools safeguard patient safety through actionable alerts
El Arab et al. [10]	Multinational	Integrative review	Secondary evidence	25 studies	Multiple AI types (CDSS, NLP, robotics, predictive analytics)	Nursing practice (clinical, educational, operational)	NAIIR 6‐pillar framework proposed for organizational AI adoption; AI improves clinical decision support, education, and workload; ethical and operational readiness gaps identified	A: AI must be applied within structured frameworks guided by clinical nursing expertise; I: organizational and individual AI integration readiness is essential
Namdar Areshtanab et al. [11]	Iran	Descriptive correlational	Primary evidence	389 nurses (Tabriz)	AI readiness and acceptance tools	Hospital (general)	41.1% had low AI knowledge; 94.3% received no AI training; positive attitude but moderate acceptance; the training gap is a critical barrier	U: limited AI knowledge leads to poor understanding of limitations; I: training and AI literacy programs are urgently needed for safe integration
Taie and Ali [12]	Egypt	Descriptive cross‐sectional	Primary evidence	250 nurses (Fayoum University Hospitals)	AI knowledge and perception tools (questionnaire‐based)	ICU, OR, ER, CCU, dialysis units	58% had unsatisfactory AI knowledge; 64.8% had a positive perception of AI challenges; significant positive correlation between knowledge level and perception (*p* = 0.009)	U: knowledge deficits impair nurses’ ability to understand AI limitations; I: educational interventions are essential to build AI readiness and safe bedside integration
Bodur et al. [13]	Turkey	Qualitative descriptive (semistructured interviews)	Primary evidence	25 nurses (Istanbul hospitals)	AI in nursing practice (broad)	Multiple hospital units (ICU, wards, OR)	Four themes identified: AI concepts; opportunities (workload reduction, relational nursing); threats (job displacement, dehumanization, over‐reliance); ethical concerns (accountability ambiguity, data privacy, cybersecurity)	R: ethical and legal accountability gaps must be recognized at the bedside; S: patient safety risks from AI malfunction and over‐reliance demand human oversight
Mohammed et al. [14]	Multinational	Systematic review	Secondary evidence	17 studies	AI ethics in nursing (broad)	Multiple nursing settings	Five themes: decision‐making enhancement, ethical awareness, task support, algorithmic bias, public engagement; gap in nursing‐specific ethical guidelines; bias and accountability risks underreported	R: Algorithmic bias and ethical accountability must be recognized by bedside nurses; S: patient safety is jeopardized without ethical oversight of AI outputs

*Note:* Evidence‐based principles and bedside applications are indicative rather than exhaustive. Framework components are intended for use as an integrated cognitive tool, not as sequential steps.

Abbreviations: CDSS, clinical decision support system; ML, machine learning; NLP, natural language processing.

Fourteen sources met the eligibility criteria. Although this limited the number of directly included publications, the sources represented diverse study designs, settings, and geographic contexts. Several included reviews also synthesized broader bodies of primary evidence. Nevertheless, the framework should be interpreted as evidence‐informed rather than as a definitive or evidence‐graded clinical tool.

### 3.3. Thematic Synthesis

Inductive thematic analysis of the 14 included studies identified five overarching themes: (1) AI applications and clinical outcomes; (2) nurses’ knowledge, attitudes, and acceptance of AI; (3) ethical and accountability challenges; (4) barriers and facilitators to AI implementation; and (5) AI literacy and training gaps. These themes directly informed the development and component mapping of the NAVIGATE‐AI cognitive guide.

The framework development occurred in four stages:

 Stage 1: identification of common themes by using inductive thematic synthesis.
 Stage 2: extraction of evidence‐based principles underpinning safe AI implementation from each theme.
 Stage 3: mapping of principles against nursing AI competency literature, patient safety principles, and healthcare AI ethics guidance.
 Stage 4: iterative grouping and refinement of principles into a mnemonic structure designed to maximize bedside usability and cognitive recall.

#### 3.3.1. Theme 1: AI Applications and Clinical Outcomes

The included studies identified a broad range of AI applications across multiple nursing domains. Park et al. [7] synthesized evidence from 35 studies in critical care and identified six AI application domains: patient monitoring, nursing interventions, CDSS, documentation, resource allocation, and predictive analytics, with predictive analytics most prevalent. Notably, narrowly focused AI applications consistently demonstrated superior clinical performance over general‐purpose tools, a distinction with direct implications for safe bedside use.

Mikkonen et al. [9] synthesized eight interventional studies (*n* = 1173) across the United States, South Korea, and Canada, confirming that AI‐supported decision‐making tools, including CDSS and machine learning–based early warning systems, significantly improved outcomes, reducing hospital readmissions, ICU transfers, and protocol deviations. Workflow integration and usability emerged as the primary determinants of whether AI tools delivered their intended clinical benefit.

Alqaraleh et al. [8] demonstrated that AI‐powered CDSS reduced operating room medication errors by up to 95%, and smart infusion pump technology reduced intravenous administration errors by up to 80%. Despite these gains, 62% of nurses reported uncertainty about trusting AI‐generated recommendations, underscoring the persistent gap between AI efficacy and nurse acceptance.

At the broader synthesis level, Khan Rony et al. [2] and Wei et al. [1] confirmed that AI technologies hold potential to enhance clinical decision support, optimize workflows, and reduce workload, yet noted that the evidence base remains heterogeneous and most gains are not yet universally scalable. O’Connor et al. [5] found that only 7.14% of 140 included studies demonstrated actual real‐world clinical benefit, a finding echoed by Seibert et al. [4], who reported that 83.2% of AI applications in nursing remained at basic or experimental stages.

#### 3.3.2. Theme 2: Nurses’ Knowledge, Attitudes, and Acceptance of AI

A consistent pattern across survey‐based studies was the significant gap between nurses’ positive attitudes toward AI and their actual AI literacy. Namdar Areshtanab et al. [11] reported a mean knowledge score of 8.89 (SD = 5.31) on a 0–16 scale among 389 nurses, with 41.1% demonstrating low AI knowledge and 94.3% having received no prior AI training. Despite limited knowledge, acceptance and attitude were positively correlated with knowledge. Because the underlying study was cross‐sectional, these associations do not establish that education improves acceptance of or safe use of AI.

Taie and Ali [12] reported knowledge deficits among nursing personnel and identified limited AI education as a perceived barrier; however, the cross‐sectional design does not establish a primary explanatory factor. A significant positive correlation was identified between knowledge and perception scores (*r* = 0.164, *p* = 0.009), supporting the inference that improved knowledge positively shifts perceptions. Bodur et al. [13] further revealed nuanced views among nurses encompassing four thematic domains: AI concepts; opportunities (workload reduction, efficiency); threats (job displacement, over‐reliance, dehumanization); and ethical and legal concerns (accountability ambiguity, data privacy, cybersecurity).

#### 3.3.3. Theme 3: Ethical and Accountability Challenges

Ethical considerations emerged as a critical and recurring theme. Mohammed et al. [14] synthesized 17 studies and identified five ethical themes: decision‐making enhancement, ethical awareness, task support, algorithmic bias, and public engagement, highlighting a critical gap in nursing‐specific ethical guidelines. von Gerich et al. [3] reported that 36.6% of included AI studies lacked any ethical discussion, and Seibert et al. [4] confirmed that ethical, legal, and social implications of AI in nursing were rarely addressed.

Both El Arab et al. [6] and El Arab et al. [10] highlighted persistent concerns regarding data privacy, algorithmic bias, and AI‐perpetuated health disparities. The black‐box opacity of advanced AI systems was specifically identified as fundamentally undermining nurses’ capacity to critically evaluate AI‐generated outputs. Bodur et al. [13] identified accountability ambiguity—the absence of clear frameworks defining professional responsibility when AI systems produce erroneous outputs—as a primary source of nurse anxiety, underscoring the ethically untenable position in which nurses currently operate.

#### 3.3.4. Theme 4: Barriers and Facilitators to AI Implementation

Barriers to AI implementation were consistently identified across technical, educational, organizational, and relational dimensions. Wei et al. [1] categorized these into technical constraints (data heterogeneity, interoperability failures, algorithmic opacity), ethical dilemmas (accountability, consent, bias), and workforce gaps (insufficient training and AI literacy), all of which represent barriers that interact in mutually reinforcing ways. O’Connor et al. [5] additionally identified poor‐quality digital health datasets as a structural barrier, noting that nurses’ limited involvement in AI system design compounds both the problem and its consequences.

Facilitators identified across the literature included participatory design approaches [10], structured AI training programs [11], transparent algorithmic design [6], and robust institutional data infrastructure [10]. Park et al. [7] emphasized that the specialized focus of AI tools as opposed to general‐purpose applications was a consistent predictor of clinical utility and nurse trust.

#### 3.3.5. Theme 5: AI Literacy and Training Gaps

The synthesis identified recurring AI literacy and training gaps across the included studies; however, the extent and nature of these gaps varied by setting, country, and study population. Namdar Areshtanab et al. [11] demonstrated that 94.3% of nurses had received no AI training, and multiple linear regression confirmed that high AI knowledge, positive attitude, and high acceptance, not demographic variables such as age or experience, were the significant predictors of AI application (*R* ^2^ = 0.53). This association suggests that artificial intelligence education may be an important contributor to nurses’ readiness to engage with AI‐supported tools; however, the cross‐sectional nature of the underlying evidence does not establish causality or identify education as the sole determinant of safe AI use.

Taie and Ali [12] confirmed that the absence of AI content in nursing curricula was the primary explanatory factor for identified knowledge deficits. El Arab et al. [6] concluded from 18 reviews that the current nursing education system inadequately prepares graduates to navigate AI‐integrated environments and that curriculum reform incorporating AI literacy, data ethics, and critical appraisal of AI outputs is urgently required. Mikkonen et al. [9] further noted a geographic bias toward US‐based evidence, necessitating caution when extrapolating implementation guidance across diverse healthcare systems.

### 3.4. Development of the NAVIGATE‐AI Nursing Framework

Drawing on the five thematic domains identified through synthesis of the 14 included studies, the NAVIGATE‐AI nursing framework was developed as a point‐of‐care tool to support bedside nurses in safe, critically grounded, and ethically responsible navigation of AI technologies. Each component of the NAVIGATE‐AI framework was mapped to a specific, evidence‐derived principle essential to safe AI use in nursing (Table 2).

**TABLE 2:** NAVIGATE‐AI nursing framework mnemonic: components, evidence‐based principles, and supporting studies.

Letter	Principle	Evidence‐based rationale	Bedside application	Key supporting studies
N	Navigate AI outputs critically	AI‐generated outputs require critical appraisal, as most evidence remains at experimental or developmental stages and AI recommendations may be inaccurate, outdated, or inapplicable to individual patient contexts	Question AI alerts: “does this output align with my clinical assessment?”	Khan Rony et al. [2]; Mikkonen et al. [9]; von Gerich et al. [3]
U	Understand AI limitations and biases	Nurses must understand the scope, boundaries, and potential biases of each AI tool used, as specialized AI consistently outperforms general tools and biased training data can perpetuate care inequities	Complete AI literacy training, review institutional AI guidance, and seek support when unfamiliar AI tools are introduced into practice	Namdar Areshtanab et al. [11]; Park et al. [7]; Taie and Ali [12]
R	Recognize ethical and accountability responsibilities	Nurses should remain attentive to professional accountability, local policy, documentation requirements, algorithmic bias, and privacy considerations when using AI‐supported recommendations	Maintain personal accountability; document AI‐assisted decisions	von Gerich et al. [3]; O’Connor et al. [5]; Bodur et al. [13]; Mohammed et al. [14]; Seibert et al. [4]; El Arab et al. [6]
S	Safeguard patient safety and human oversight	AI tools should not replace nurse oversight; in higher‐risk clinical contexts, nurses should consider whether independent clinical verification is needed before acting on AI‐supported recommendations	Override AI recommendations when clinical signs contradict AI outputs	Bodur et al. [13]; Park et al. [7]; Alqaraleh et al. [8]; Mohammed et al. [14]
E	Evaluate evidence before applying AI recommendations	Nurses must assess the quality, clinical context, and external validity of AI recommendations before incorporating them into clinical decisions, given that many AI tools reported in the included literature had limited real‐world clinical evaluation or uncertain transferability across settings	Verify the AI recommendation against current clinical guidelines	von Gerich et al. [3]; O’Connor et al. [5]; Alqaraleh et al. [8]; Seibert et al. [4]
A	Apply AI with clinical judgment	AI outputs should function as one input among many in clinical reasoning; clinical judgment, patient values, and contextual knowledge must always remain primary determinants of nursing decisions	Use AI as a second opinion, not a replacement for nursing assessment	Khan Rony et al. [2]; Wei et al. [1]; El Arab et al. [10]; Mikkonen et al. [9]
I	Integrate AI literacy and readiness into nursing practice	Safe and informed AI use may be supported by foundational AI literacy, including awareness of algorithmic limitations, bias, data ethics, and local governance requirements	Before applying the AI output, ask ‘was this tool validated in a patient population similar to mine?’ Consult the tool documentation or institutional guidelines	Namdar Areshtanab et al. [11]; Taie and Ali [12]; Wei et al. [1]; El Arab et al. [6]; El Arab et al. [10]

*Note:* The framework is evidence‐informed and was subsequently subjected to an initial expert content evaluation. It is intended as an integrated cognitive aid and should not be interpreted as a validated clinical decision rule or a substitute for professional judgment, local policy, or institutional governance.

### 3.5. Expert Content Evaluation

Of the 32 questionnaires received, one duplicate response was excluded, leaving 31 analyzable responses. The self‐reported characteristics of the expert panel are presented in Table 3. Most respondents reported more than 3 years of clinical or academic experience (*n* = 27, 87.1%). The panel represented self‐reported expertise in nursing informatics, artificial intelligence in healthcare, oncology nursing, patient safety, healthcare ethics, critical care nursing, and wound management or vascular access device care.

**TABLE 3:** Self‐reported characteristics of the expert panel included in the initial content evaluation (*n* = 31).

Characteristic	*n* (%)
Questionnaires received	32
Duplicate responses excluded	1
Analyzable responses	31
Highest nursing/academic qualification	
BSN	15 (48.4)
MSN	8 (25.8)
PhD	6 (19.4)
RN	2 (6.5)
Professional role reported	
Registered nurse	1 (3.2)
Nurse informatics	1 (3.2)
Not reported	29 (93.5)
Years of clinical/academic experience	
More than 3 years	27 (87.1)
1–3 years	3 (9.7)
Not reported	1 (3.2)
Self‐reported area of expertise	
Nursing informatics	10 (32.3)
Artificial intelligence in healthcare	8 (25.8)
Oncology nursing	5 (16.1)
Patient safety	5 (16.1)
Healthcare ethics	2 (6.5)
Critical care nursing	1 (3.2)
Wound management/vascular access device care	1 (3.2)
Country/institution reported	
Jordan	24 (77.4)
Egypt	6 (19.4)
Not reported	1 (3.2)

*Note:* Characteristics were self‐reported in an anonymous online questionnaire. Educational qualifications and professional roles are reported separately. Percentages do not sum to 100% for the area of expertise because respondents could report more than one area. The country or institution was not reported by all respondents. Two respondents reported registered nurse (RN) licensure without specifying an academic degree; these were retained as a separate category to avoid assuming a specific level of academic qualification.

Relevance I‐CVI values for the seven framework components ranged from 0.68 to 0.94 (Table 4). The S‐CVI/Ave was 0.78, which was below the prespecified reference threshold of 0.90. Clarity ratings of 3 or 4 ranged from 83.9% to 96.8%, and perceived bedside applicability ratings of 3 or 4 ranged from 87.1% to 96.8%. Overall, 25 of 31 respondents (80.6%) rated the framework as suitable or highly suitable for point‐of‐care use. These findings provide preliminary evidence regarding relevance, clarity, and perceived bedside applicability; however, they do not establish content validity at scale, clinical validity, usability, feasibility, implementation effectiveness, or patient benefit.

**TABLE 4:** Initial expert content evaluation of the NAVIGATE‐AI nursing framework (*n* = 31).

Component	Relevance, *n*/*N*	Clarity: rating 3 or 4, *n* (%)	Applicability: rating 3 or 4, *n*(%)	Action after expert review
Navigate AI outputs critically	25/31	26 (83.9)	27 (87.1)	Wording retained; critical appraisal before action emphasized
Understand limitations and potential biases	21/31	28 (90.3)	29 (93.5)	Wording retained
Recognize ethical and accountability responsibilities	22/31	27 (87.1)	28 (90.3)	Accountability language clarified
Safeguard patient safety through human oversight	29/31	30 (96.8)	30 (96.8)	Wording retained
Evaluate evidence supporting AI recommendations	24/31	29 (93.5)	29 (93.5)	Wording retained
Apply AI as a complement to clinical judgment	26/31	28 (90.3)	29 (93.5)	Complementary role of AI emphasized
Integrate AI literacy into routine nursing practice	23/31	28 (90.3)	28 (90.3)	Wording retained
Scale‐level content validity index, average method (S‐CVI/Ave)	0.78	—	—	—

*Note:* I‐CVI = item‐level content validity index; S‐CVI/Ave = scale‐level content validity index using the average method. The S‐CVI/Ave of 0.78 was below the prespecified reference threshold of 0.90. Accordingly, the findings are interpreted as preliminary expert content‐evaluation findings, not as confirmation of scale‐level content validity. Wording refinements informed by expert feedback were not subjected to a second round of rating.

These results provide preliminary evidence regarding the relevance, clarity, and perceived bedside applicability of the proposed components. However, they do not establish full scale‐level content validity according to the prespecified S‐CVI/Ave threshold, nor do they establish clinical validity, usability, feasibility, implementation effectiveness, or patient benefit. Free‐text comments were reviewed descriptively and informed minor wording clarification. Because the revised wording was not rerated in a subsequent round, further iterative expert consensus evaluation is needed.

As a sensitivity analysis, relevance I‐CVI values were recalculated for the subgroup of respondents who reported direct expertise in artificial intelligence in healthcare or nursing informatics (*n* = 18, 58.1%). Within this subgroup, I‐CVI values ranged from 0.61 to 0.89: Navigate, 0.72; understand, 0.72; recognize, 0.61; safeguard, 0.89; evaluate, 0.72; apply, 0.83; and integrate, 0.72. The subgroup S‐CVI/Ave was 0.75. This pattern was broadly consistent with the full‐sample findings and did not indicate more favorable ratings among respondents with direct AI/informatics expertise.

The overall S‐CVI/Ave was 0.78, which was below the prespecified reference threshold of 0.90. Therefore, the findings should be interpreted as preliminary evidence of item relevance and perceived bedside applicability rather than confirmation of scale‐level content validity.

### 3.6. Framework Development Methodology

The framework was developed using a structured evidence‐to‐framework approach based on implementation science and knowledge translation methods. After thematic synthesis, recurring concepts were thoroughly reviewed by the research team and translated into candidate domains. Domains were iterated through comparison with existing nursing AI competency frameworks, patient safety principles, and AI ethics guidance. Domains with overlapping concepts were aggregated to produce a seven‐domain model.

Based on thematic synthesis of 14 included studies, five overarching themes were identified. Evidence‐based principles specific to safe bedside AI implementation were extracted and consolidated into seven domains. These domains were later organized into the NAVIGATE‐AI nursing framework to provide nurses with a structured cognitive aid supporting safe, ethical, and evidence‐informed use of artificial intelligence in clinical practice. A visual summary facilitating rapid bedside recall is presented in Figure 2.

**FIGURE 2:**
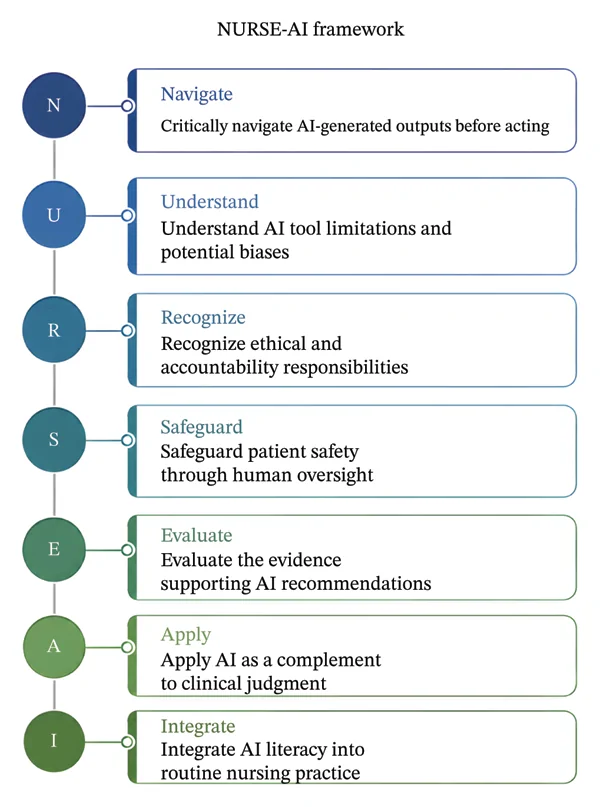
The NAVIGATE‐AI nursing framework.

The framework components are intended as an integrated cognitive aid rather than sequential clinical steps, a validated clinical decision rule, or a substitute for professional judgment and local governance.

## 4. Discussion

### 4.1. Principal Findings in Context

This scoping review synthesized evidence from 14 sources published between 2021 and 2026, mapping AI applications, nurse–AI interactions, and implementation challenges in nursing clinical practice. The findings suggest that AI may support clinical decision‐making, patient monitoring, medication safety, and workflow efficiency; however, they also reveal substantial gaps between AI’s potential and its safe, equitable, and evidence‐based implementation at the bedside.

The most striking finding concerns the divergence between AI efficacy data and real‐world clinical implementation. While O’Connor et al. [5] found that only 7.14% of 140 AI studies demonstrated actual real‐world clinical benefit, and von Gerich et al. [3] reported that 93% of AI technologies remained in developmental phases (through 2021), studies from 2025 to 2026 already reported AI‐driven reductions in medication errors of up to 95% [8] and measurable improvements in clinical decision‐making outcomes [9]. These findings suggest that AI applications in nursing are evolving rapidly; therefore, structured support for nurses’ critical appraisal and safe use of AI‐supported recommendations should be considered alongside local implementation and governance processes.

### 4.2. AI Literacy and Workforce Preparation

The included studies identified important gaps in knowledge and training in artificial intelligence among nursing participants. In one Iranian hospital‐based cohort, 94.3% of participating nurses reported no prior artificial intelligence training, whereas a study of Egyptian nursing personnel found that 58% had unsatisfactory artificial intelligence knowledge [11, 12]. These context‐specific findings should not be interpreted as prevalence estimates for nurses globally. Nevertheless, together with findings from the included evidence syntheses, they suggest that artificial intelligence literacy may be an important area for nursing education and professional development [2, 6, 10].

This concern is compounded by evidence that AI deployment is advancing far ahead of nursing workforce preparedness. Alqaraleh et al. [8] demonstrated that despite proven AI efficacy in medication safety, 62% of nurses remained uncertain about trusting AI recommendations, a paradox creating dual risk: distrust of effective AI may cause nurses to miss valid alerts, while uncritical acceptance of erroneous AI outputs may prompt unsafe clinical actions. The NAVIGATE‐AI nursing framework ‘U’ (understand AI limitations) and ‘I’ (integrate AI literacy) components directly address this duality through a scaffolded approach to critical AI engagement. Importantly, in the regression analysis by Namdar Areshtanab et al. [11] in the cited Iranian hospital‐based study, AI knowledge, attitude, and acceptance were associated with AI application; however, the cross‐sectional design does not establish causality or determine the optimal scope, format, or target population for training.

### 4.3. From Efficacy Data to Bedside Safety: The Critical Appraisal Imperative

A clinically significant finding across the synthesis was that AI performance in controlled development environments does not reliably predict performance in real‐world deployment. von Gerich et al. [3] documented substantial deterioration in AI performance under real‐world multisite conditions compared to developmental phases, exemplified by proprietary sepsis prediction models, underscoring the imperative of contextual evaluation. This example illustrates that evidence supporting an AI tool’s performance in one context cannot be assumed to transfer to a different patient population, EHR system, or clinical workflow, a principle operationalized in the NAVIGATE‐AI nursing framework ‘N’ (navigate AI outputs critically) and ‘E’ (evaluate evidence) components.

Park et al. [7] demonstrated that specialized AI tools consistently outperform general‐purpose applications in clinical nursing contexts, and Mikkonen et al. [9] identified usability and workflow integration, not algorithmic accuracy, as the primary determinants of whether AI decision‐support tools delivered clinical benefit. AI tools that fit naturally within the nursing workflow generated actionable outputs and produced measurable improvements; technically accurate tools that disrupted workflow or contributed to alert fatigue failed to deliver benefit regardless of algorithmic performance. This finding directly supports the framework’s ‘A’ (apply AI with clinical judgment) component.

### 4.4. Ethical and Governance Considerations for Patient Safety

The ethical dimensions of AI in nursing emerged not as abstract concerns, but as concrete patient safety risks. Mohammed et al. [14] identified algorithmic bias, lack of AI transparency, and the absence of nursing‐specific ethical guidelines as the three most pressing ethical challenges, findings convergent with von Gerich et al. [3], who documented systematic ethical omissions across the AI nursing research literature, including a complete absence of ethical discussion at the implementation and operational phases.

The black‐box opacity of advanced AI creates a specific accountability paradox: when a nurse acts on an AI‐supported recommendation without sufficient understanding of its intended use, limitations, or local governance requirements, questions regarding professional accountability may arise and warrant clear institutional guidance. Bodur et al. [13] confirmed this concern among Turkish hospital nurses, who expressed significant anxiety about being held professionally accountable for outcomes arising from AI recommendations they lacked the knowledge to evaluate. The NAVIGATE‐AI nursing framework ‘R’ (recognize ethical and accountability responsibilities) component operationalizes the principle that professional accountability in nursing cannot be delegated to an algorithm. El Arab et al. [6] further proposed that robust ethical governance requires comprehensive nursing‐specific frameworks, integration of AI ethics into curricula, and equitable access to AI technologies; these recommendations are reflected across multiple NAVIGATE‐AI components.

### 4.5. The NAVIGATE‐AI Nursing Framework in the Landscape of Existing Nursing AI Frameworks

The NAVIGATE‐AI nursing framework makes a distinct contribution to the existing landscape of nursing AI tools. El Arab et al. [10] proposed the NAIIR, a six‐pillar framework for organizational‐level AI adoption; however, NAIIR is not designed to support individual nurses at the point of care. Similarly, El Arab et al. [6] advanced the case for curriculum reform at the population level, and Khan Rony et al. [2] and Wei et al. [1] identified the need for nursing‐specific AI tools without generating a practical bedside implementation instrument. A related mnemonic, N.U.R.S.E.S., was proposed in 2025 to support artificial intelligence literacy among nurses at the bedside [18]. The present framework should therefore be interpreted as complementary to, rather than superior to or a replacement for, existing nursing AI guidance. Comparative usability, educational effectiveness, and implementation studies are required to determine whether NAVIGATE‐AI offers added value beyond existing AI literacy and organizational implementation frameworks. The updated literature search conducted through September 2026 did not identify additional nursing‐specific AI frameworks published after the original search beyond NAIIR and N.U.R.S.E.S.; the present framework is therefore positioned relative to these two existing approaches.

NAVIGATE‐AI is proposed as a complementary bedside‐oriented mnemonic within an emerging landscape of nursing AI literacy, implementation, governance, and clinical practice frameworks. Its contribution is not that it is the first nursing‐focused AI mnemonic; rather, it translates themes from the present scoping review into a seven‐component cognitive aid and reports preliminary findings from expert content evaluation. Its seven components map directly onto the five thematic domains identified in the synthesis, ensuring firm grounding in the empirical evidence base rather than theoretical first principles alone. The mnemonic format is deliberate and evidence‐informed: mnemonic frameworks have a well‐established role in high‐stakes clinical settings from basic life support algorithms to sepsis bundles and surgical safety checklists [19, 20] precisely because they provide structured cognitive scaffolding under conditions of time pressure and information overload. Given that AI technologies are most commonly deployed in high‐acuity, time‐sensitive environments [7, 9], a mnemonic format is particularly well‐suited to support safe AI engagement in the moments that matter most. This need is especially relevant in critical care, where expanding AI use continues to be limited by challenges in evaluation, interpretability, interoperability, and ethical governance [21].

## 5. Implications for Clinical Practice and Future Research

### 5.1. Implications for Nursing Management

Nurse managers and health‐system leaders play a central role in ensuring the safe integration of AI at the bedside. Key management considerations arising from this review include the following:

Governance: establish clear institutional policies defining when AI‐supported recommendations require independent clinical verification and designate accountable roles for AI‐related decision oversight.

Competency development: prioritize structured, role‐appropriate AI literacy training as an ongoing professional development requirement rather than a one‐time orientation activity.

Procurement: involve nursing leadership in AI tool procurement decisions, evaluating intended use, patient population fit, workflow integration, and available evaluation evidence before adoption [3–5, 7–9].

Escalation pathways: develop and communicate clear procedures for nurses to report suspected AI malfunction, override erroneous AI recommendations, and escalate concerns without fear of professional repercussion.

Monitoring: implement ongoing monitoring of AI tool performance post‐deployment, including tracking of near‐misses, overrides, and alert‐fatigue indicators.

Accountability: clarify professional accountability structures when AI‐supported recommendations contribute to adverse outcomes, ensuring nurses are not held solely accountable for system‐level AI limitations.

Nurse involvement: formally include bedside nurses in AI system codesign, pilot testing, and evaluation to improve workflow fit and adoption.

These management considerations are derived from the evidence synthesis and the authors’ professional judgment; they represent practice recommendations rather than direct empirical findings, and their effectiveness requires prospective evaluation.

### 5.2. Implications for Future Research

Longitudinal and interventional studies are urgently needed to address the fundamental limitation identified by O’Connor et al. [5] and Seibert et al. [4]: the paucity of studies demonstrating actual clinical benefit in real‐world settings. Future research should employ prospective, interventional designs, including stepped‐wedge cluster trials and pragmatic RCTs to evaluate AI tools’ impact on patient safety outcomes, workflow efficiency, and nurse burnout across diverse healthcare systems.

Geographic diversification of the nursing AI evidence base is essential, given the significant skew toward US‐based healthcare settings documented by Mikkonen et al. [9]. Research in low‐ and middle‐income countries and systems with different nursing education standards and patient population profiles is necessary to ensure that global AI governance frameworks are genuinely generalizable. Rigorous evaluation of the NAVIGATE‐AI nursing framework itself is a priority: mixed‐methods studies should assess nurses’ uptake and usability perceptions, examine whether NAVIGATE‐AI–guided engagement improves decision‐making quality, and evaluate its educational impact when integrated into nursing curricula.

Development of nursing‐specific AI ethical frameworks and regulatory guidelines represents another critical gap. Mohammed et al. [14] and El Arab et al. [6] documented that existing frameworks fail to account for the specific accountability structures and practice contexts of nursing. Focused research on alert fatigue and AI over‐reliance, together with investigation of patient and family perspectives on AI‐assisted nursing care, represents a substantially under‐researched area that is essential to ensuring AI integration enhances rather than undermines patient‐centered, compassionate nursing care.

### 5.3. Limitations

Several limitations should be considered. First, the review included heterogeneous primary studies and secondary evidence syntheses and overlap of primary studies across the included reviews was not formally quantified. Accordingly, the findings should not be interpreted as an independent count of cumulative evidence or as a graded estimate of intervention effectiveness. Second, no formal risk‐of‐bias assessment was undertaken, consistent with the mapping purpose of scoping reviews [15–17]. Organizational gray‐literature searching (WHO, ICN, ANA) identified 27 records, and backward/forward citation tracking identified 34 records (61 records total from these supplementary streams). After screening, 6 reports were assessed at full text; none met the prespecified peer‐reviewed eligibility criteria for final inclusion. To reduce the influence of isolated findings during framework development, each component was informed by convergent evidence from multiple included sources rather than a single source. Third, the expert content evaluation used a purposive and predominantly Jordan‐based sample, and the S‐CVI/Ave of 0.78 did not meet the prespecified reference threshold of 0.90. Fourth, the anonymous survey design prevented confirmation of whether respondents had prior involvement in framework development, and wording refinements after expert feedback were not re‐rated in a second round. Finally, the study did not evaluate clinical usability, feasibility, workflow integration, implementation outcomes, or patient outcomes. Further iterative consensus work and prospective evaluation are required before broad application of the framework. An additional limitation is that registration occurred after the review process had commenced, and the final methods differed from the registered protocol through the inclusion of narrative reviews, the clarification of exclusion criteria during full‐text screening, and the addition of a subsequent expert content‐evaluation exercise.

## 6. Conclusion

This scoping review mapped heterogeneous evidence on artificial intelligence applications, nurse–AI interactions, ethical considerations, implementation barriers, and artificial intelligence literacy in nursing clinical practice. The NAVIGATE‐AI nursing framework was developed as an evidence‐informed, bedside‐oriented mnemonic that translates recurring principles into seven components intended to support critical, safe, and ethically attentive engagement with AI‐supported recommendations. The initial expert content evaluation showed generally favorable item‐level ratings for relevance, clarity, and perceived bedside applicability. However, the S‐CVI/Ave of 0.78 was below the prespecified reference threshold of 0.90. Accordingly, the findings should be interpreted as preliminary evidence of content relevance rather than confirmation of scale‐level content validity or clinical effectiveness.

## Author Contributions

O.A. conceived the study, designed the search strategy, conducted screening, extracted data, and drafted the manuscript. S.D. contributed to data extraction, participated in screening, and critically revised the manuscript. M.D. contributed to the thematic analysis and revised the manuscript for intellectual content. S.A‐G. supervised the review methodology and reviewed the manuscript.

## Funding

The authors received no specific funding for this work.

## Disclosure

All authors approved the final version and agreed to be accountable for all aspects of the work.

## Ethics Statement

Ethical approval was not required for the scoping review component because it involved the analysis of previously published, publicly available evidence and did not involve patient‐level or human‐subject data. For the expert content‐evaluation exercise, the authors did not seek formal institutional ethics board review, consistent with common institutional practice for anonymous, voluntary professional‐opinion surveys that collect no sensitive personal data or identifiable information and pose no more than minimal risk to participants. This determination was made by the research team based on the nature of the data collected rather than through a formal institutional ethics committee ruling. Participation was voluntary, responses were collected anonymously without identifying information, and completion of the questionnaire was considered evidence of informed consent. No incentives were offered.

## Consent

Informed consent was implied by voluntary completion and submission of the anonymous expert content‐evaluation questionnaire.

## Conflicts of Interest

The authors declare no conflicts of interest.

## Supporting Information

Additional supporting information can be found online in the Supporting Information section.

## Supporting information


**Supporting Information** The supporting information includes three tables. Table S1 provides the detailed search strategies and search dates for the databases and organizational websites consulted. Table S2 presents the evidence‐to‐framework derivation matrix, linking the review themes and extracted practice principles to the NAVIGATE‐AI nursing framework components and refinements following expert feedback. Table S3 contains the expert content‐evaluation questionnaire used to assess the framework’s relevance, clarity, applicability, and overall suitability for bedside use.

## Data Availability

The deidentified expert content‐evaluation dataset and the questionnaire instrument are available from the corresponding author upon reasonable request. Database‐specific search strategies are provided in Supporting Table S1, the evidence‐to‐framework derivation matrix is provided in Supporting Table S2, and the expert content‐evaluation questionnaire is provided in Supporting Table S3.
