# Supplementary material for: NAVIGATE‐AI Nursing Framework: A Scoping Review and Preliminary Expert Content Evaluation for Safe Bedside AI Use in Nursing
Source: J Nurs Manag. 2026 Oct 9;2026:6525145. doi: 10.1155/jonm/6525145 (PMC13653144; doi:10.1155/jonm/6525145)
Supplement: Supplementary file 1 [file JONM-2026-6525145-s001.docx]

**Table S1.** Detailed Search Strings by Database

**Search Period:** January 2015 to September 2026

| **Database** | **Search String** | **Date Searched** |
| --- | --- | --- |
| PubMed / MEDLINE | ("artificial intelligence"[MeSH Terms] OR "machine learning"[MeSH Terms] OR "deep learning"[tiab] OR "natural language processing"[MeSH Terms] OR "clinical decision support systems"[MeSH Terms] OR "predictive analytics"[tiab] OR "neural network"[tiab] OR "algorithm"[tiab]) AND ("nursing"[MeSH Terms] OR "nurses"[MeSH Terms] OR "nursing practice"[tiab] OR "nursing care"[tiab] OR "bedside nursing"[tiab] OR "clinical nursing"[tiab] OR "nurse"[tiab]) AND ("implementation"[tiab] OR "integration"[tiab] OR "clinical practice"[tiab] OR "patient safety"[MeSH Terms] OR "patient outcomes"[tiab] OR "adoption"[tiab] OR "barriers"[tiab] OR "knowledge"[tiab] OR "attitudes"[tiab]) | September 2026 |
| CINAHL (via EBSCOhost) | (MH "Artificial Intelligence" OR MH "Machine Learning" OR TI "deep learning" OR AB "deep learning" OR MH "Natural Language Processing" OR TI "predictive analytics" OR AB "predictive analytics" OR TI "clinical decision support" OR AB "clinical decision support") AND (MH "Nursing Practice" OR MH "Nurses" OR TI "nursing" OR AB "nursing" OR TI "bedside nurse*" OR AB "bedside nurse*" OR TI "clinical nurs*" OR AB "clinical nurs*") AND (TI "implement*" OR AB "implement*" OR TI "integrat*" OR AB "integrat*" OR TI "patient safety" OR AB "patient safety" OR TI "barrier*" OR AB "barrier*" OR TI "facilitator*" OR AB "facilitator*" OR TI "knowledge" OR AB "knowledge" OR TI "attitude*" OR AB "attitude*") | September 2026 |
| Scopus | (TITLE-ABS-KEY("artificial intelligence" OR "machine learning" OR "deep learning" OR "natural language processing" OR "predictive analytics" OR "clinical decision support" OR "neural network*") AND TITLE-ABS-KEY("nursing" OR "nurse*" OR "nursing practice" OR "nursing care" OR "bedside nursing" OR "clinical nursing") AND TITLE-ABS-KEY("implementation" OR "integration" OR "clinical practice" OR "patient safety" OR "patient outcomes" OR "adoption" OR "barriers" OR "knowledge" OR "attitudes" OR "ethical*" OR "framework*")) AND PUBYEAR > 2014 AND LANGUAGE(english) | September 2026 |
| ScienceDirect | ("artificial intelligence" OR "machine learning" OR "deep learning" OR "natural language processing" OR "predictive analytics" OR "clinical decision support") AND ("nursing" OR "nurse" OR "nursing practice" OR "nursing care" OR "bedside nursing") AND ("implementation" OR "integration" OR "patient safety" OR "barriers" OR "ethical" OR "framework") [Title, Abstract, Keywords; 2015–2026] | September 2026 |
| Web of Science | TS=("artificial intelligence" OR "machine learning" OR "deep learning" OR "natural language processing" OR "predictive analytics" OR "clinical decision support") AND TS=("nursing" OR "nurse*" OR "nursing practice" OR "nursing care" OR "bedside nurs*" OR "clinical nurs*") AND TS=("implementation" OR "integration" OR "clinical practice" OR "patient safety" OR "patient outcomes" OR "barriers" OR "facilitators" OR "knowledge" OR "attitudes" OR "ethical*" OR "framework*") AND PY=(2015-2026) AND LA=(English) | September 2026 |
| World Health Organization website | “Artificial intelligence” AND nursing; AI AND ethics; AI AND patient safety | September 2026 |
| International Council of Nurses website | “Artificial intelligence” AND nursing; AI AND competency; AI AND guidance | September 2026 |
| American Nurses Association website | “Artificial intelligence” AND nursing; AI AND ethics; AI AND guidance | September 2026 |

**Table S2**. Evidence-to-Framework Derivation Matrix for the NAVIGATE-AI Nursing Framework

| **Theme** | **Extracted practice principles** | **Supporting sources** | **Candidate domains considered** | **Rationale for consolidation** | **Final NAVIGATE-AI component** | **Refinement after expert feedback** |
| --- | --- | --- | --- | --- | --- | --- |
| AI applications and clinical outcomes | AI-supported outputs require contextual clinical appraisal; evidence of benefit may not transfer across settings; applicability and workflow fit require consideration | [2–5,9] | Critical appraisal; contextual applicability; evidence evaluation | Critical appraisal of an immediate AI output and evaluation of the evidence supporting its use were retained as related but distinct bedside tasks | N: Navigate AI outputs critically; E: Evaluate evidence supporting AI recommendations | The emphasis on appraisal before action was retained |
| Nurses’ knowledge, attitudes, and acceptance | Nurses require understanding of the scope, limitations, and potential biases of AI tools; education may support informed engagement | [7,11–13] | AI literacy; limitations; bias awareness; readiness | Tool-specific understanding was distinguished from broader ongoing AI literacy and readiness | U: Understand limitations and potential biases; I: Integrate AI literacy into routine nursing practice | Wording retained; emphasis on practical AI literacy retained |
| Ethical and accountability challenges | Nurses should consider privacy, algorithmic bias, transparency, documentation, and professional accountability when using AI-supported recommendations | [3–6,13,14] | Ethics; accountability; transparency; governance | Ethical concerns and accountability were combined because both address responsibilities associated with use of AI-supported recommendations | R: Recognize ethical and accountability responsibilities | Accountability language was clarified |
| Barriers and facilitators to implementation | Safe use depends on human oversight, workflow fit, institutional support, and nurse involvement in implementation and governance | [1,3,5,7,9,10] | Human oversight; workflow integration; governance; co-design | Human oversight was retained as an immediately actionable bedside safety domain; governance was positioned as an organizational complement | S: Safeguard patient safety through human oversight; A: Apply AI as a complement to clinical judgment | The complementary role of AI in clinical judgment was emphasized |
| AI literacy and training gaps | Nursing education may address AI concepts, bias recognition, data ethics, output interpretation, and appropriate escalation when uncertainty exists | [1,6,10–12] | Education; continuing competence; readiness; institutional support | Continuing AI literacy and readiness were combined to reflect ongoing learning within local governance structures | I: Integrate AI literacy into routine nursing practice | Wording retained |

**Note.** AI = artificial intelligence. The framework development process was conducted after thematic synthesis as an evidence-informed knowledge translation activity. The NAVIGATE-AI components are intended as an integrated cognitive aid and not as sequential clinical decision steps or a validated clinical decision rule.

**Table S3. Expert Content-Evaluation Questionnaire**

| **Section** | **Questionnaire item** |
| --- | --- |
| Participant information | Self-reported professional title, role, registration, or highest nursing/academic qualification |
| Participant information | Years of clinical/academic experience |
| Participant information | Area of expertise |
| Participant information | Country/institution |
| Relevance rating | This component is relevant to safe bedside AI implementation in nursing. |
| Clarity rating | The wording of this component is clear and understandable to a bedside nurse. |
| Applicability rating | This component can realistically be applied during direct clinical care. |
| Free-text comment | Please suggest any revision to the wording or note if this component overlaps with another. |
| Framework sequence | Is the sequence/order of the seven components logical and useful for bedside recall? |
| Framework completeness | Does the framework omit any important component related to safe AI use in nursing? |
| Framework overlap | Is there any overlap or redundancy between two or more components? |
| Overall suitability | Overall, how suitable is the framework for point-of-care use? |

Note. The questionnaire was administered when the draft framework was labeled “NURSE-AI.” The framework was subsequently renamed NAVIGATE-AI to avoid confusion with a separately published framework using the same name. The seven evaluated components, questionnaire items, and ratings were unchanged. The original questionnaire used an open-text item for professional title/position. For reporting in Table 3, responses were categorized as academic qualification, professional registration, or professional role, where applicable.
